# Effects of Mnemonic Strategy Training on Brain Activity and Cognitive Functioning of Left-Hemisphere Ischemic Stroke Patients

**DOI:** 10.1155/2019/4172569

**Published:** 2019-05-09

**Authors:** Alana X. Batista, Paulo R. Bazán, Adriana B. Conforto, Maria da Graça M. Martin, Sharon. S. Simon, Benjamin Hampstead, Eberval Gadelha Figueiredo, Eliane C. Miotto

**Affiliations:** ^1^Department of Neurology, Faculdade de Medicina FMUSP, Universidade de Sao Paulo, Sao Paulo, SP, Brazil; ^2^Department of Radiology, Faculdade de Medicina FMUSP, Universidade de Sao Paulo, Sao Paulo, SP, Brazil; ^3^Department of Psychiatry, Faculdade de Medicina FMUSP, Universidade de Sao Paulo, Sao Paulo, SP, Brazil; ^4^Department of Psychiatry and Michigan Alzheimer's Disease Center, University of Michigan, Ann Arbor, Michigan, USA

## Abstract

Memory dysfunction is one of the main cognitive impairments caused by stroke, especially associative memory. Therefore, cognitive training, such as face-name mnemonic strategy training, could be an important intervention for this group of patients. The goal of this study was to evaluate the behavioral effects of face-name mnemonic strategy training, along with the neural substrate behind these effects, in the left frontoparietal lobe stroke patients. Volunteers underwent 2 sessions of functional magnetic resonance imaging (fMRI) during face-name association task: one prior and the other after the cognitive training. The fMRI followed a block design task with three active conditions: trained face-name pairs, untrained face-name pairs, and a couple of repeated face-name pairs. Prior to each fMRI session, volunteers underwent neuropsychological assessment. Training resulted in better performance on delayed memory scores of HVLT-R, and on recognition on a generalization strategy task, as well as better performance in the fMRI task. Also, trained face-name pairs presented higher activation after training in default-mode network regions, such as the posterior cingulate cortex, precuneus, and angular gyrus, as well as in lateral occipital and temporal regions. Similarly, untrained face-name pairs also showed a nonspecific training effect in the right superior parietal cortex, right supramarginal gyrus, anterior intraparietal sulcus, and lateral occipital cortex. A correlation between brain activation and task performance was also found in the angular gyrus, superior parietal cortex, anterior intraparietal sulcus, and lateral occipital cortex. In conclusion, these results suggest that face-name mnemonic strategy training has the potential to improve memory performance and to foster brain activation changes, by the recruitment of contralesional areas from default-mode, frontoparietal, and dorsal attention networks as a possible compensation mechanism.

## 1. Introduction

The presence of cognitive deficits after stroke has been demonstrated in, at least, one-third of survivors and can continue for many years generating significant impact in their quality of life and higher mortality [1–3]. Among the main cognitive impairments following a stroke, memory dysfunction, particularly episodic and associative memory deficits, is related to problems in learning and recalling new information and associating different stimuli, such as faces and names [4–6]. Therefore, reducing the impact of these deficits after stroke is a relevant goal. In this context, cognitive training (CT) interventions have demonstrated positive benefits in patients with acquired brain lesions, including stroke, and have been recommended as standard practice [7, 8]. Recent studies have indicated significant benefits in patients with stroke after CT using repetition-lag memory training, designed to improve recollection of new information [9] and computerized CT for memory and attention impairments [10].

Despite the above studies, there has been no investigation on the effects of face-name training in patients with stroke lesions. Difficulties in recalling people's names can produce important impact on social interactions and communication. Previous studies in patients with amnestic mild cognitive impairment (aMCI), a neurodegenerative condition, using face-name mnemonic strategy training (MST), showed significant behavior improvement associated with recruitment of widespread cerebral networks including the frontoparietal, inferior parietal cortex, temporal and fusiform gyri, angular gyrus, posterior cingulate cortex, and precuneus using functional magnetic resonance imaging (fMRI) [11, 12]. MCI training gains were evident on similar measures in the same trained cognitive domain (“near-transfer effect”) [12, 13]. In addition, there were some evidence of training benefits to other cognitive tasks (“far-transfer effects”) while patients referred more satisfaction and improvement with their memory after the training sessions [13]. The face-name mnemonic strategy applied by these previous studies is a specific cognitive method that facilitates the organization and association of new information (i.e., a proper name to its respective face), thereby enhancing depth processing and encoding [14]. It is also considered an ecologically valid approach in cognitive rehabilitation and has been associated with the declarative or explicit memory system [11–13].

Therefore, the aim of the current study was to investigate the behavior effects and the neural correlates of MST in patients with chronic stroke lesions in the left hemisphere using fMRI before and after the training. We replicated similar procedures from previous studies that showed effective MST during the associative encoding of face-name pairs in MCI patients [11–13] that was previously adapted for Brazilian population [12, 15].

## 2. Materials and Methods

### 2.1. Patient Selection

**Figure 1:**
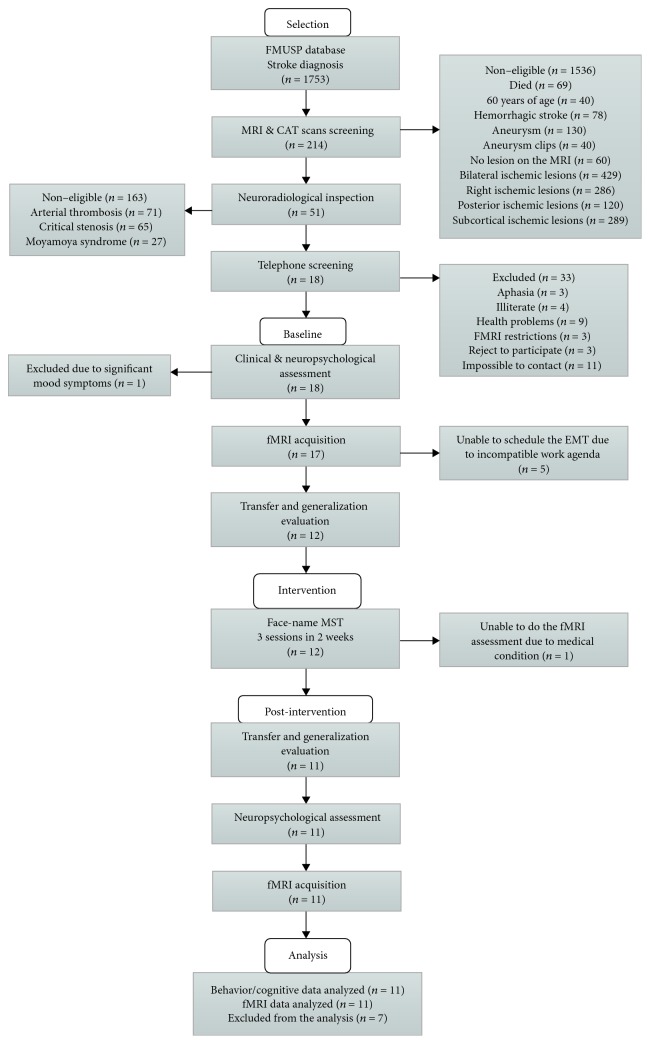
Flowchart for the selection of the stroke patients and the study design. MRI: magnetic resonance imaging; CAT: computed tomography; fMRI: function magnetic resonance imaging; and MST: mnemonic strategy training.

Eleven ischemic stroke patients who had lesions in the left frontoparietal region more than six months before fMRI scanning were recruited from a total of 1753 stroke patients of the Vascular Neurologic Clinic at the Department of Neurology, Hospital das Clinicas, Sao Paulo University database. Briefly, the patient's selection involved three hierarchical phases. First, we examined the magnetic resonance imaging (MRI) and computed tomography (CAT) scan clinical reports of patients with stroke diagnosis screened from 2009 to 2016 and selected 214 patients with ischemic lesions only in the left hemisphere without lesions on temporo-occipital, hippocampal, and bilateral lesions and critical stenosis or arterial thrombosis. Then, their MRI and/or CAT images available in the hospital database were assessed by a neuroradiologist who selected 51 left ischemic frontal stroke patients. Thereafter, we proceeded with a telephone call interview to invite them to participate and to check if they were right-handed, had no expressive or comprehension language complaints, were free from other neurological or psychiatric disorders, and had no restrictions for the fMRI exam. Eighteen nonaphasic left frontoparietal stroke patients agreed to participate in the study and signed a written informed consent prior to their inclusion in the study at the baseline evaluation. After this step, seven patients left the study due to medical problems or incompatible work schedule (see details in Section 2.2). The remaining eleven patients completed all the study steps and their data were considered at the scope of this paper. A detailed description of the patient's selection procedures is illustrated in Figure 1.

### 2.2. Procedures

**Figure 2:**
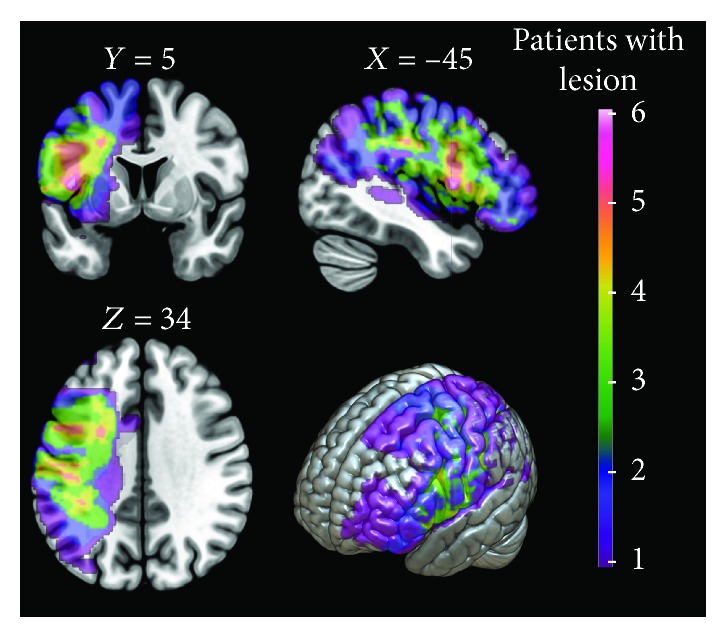
Patients' lesion map indicating the injured brain regions over the MNI152 template and how many of the 11 patients included had lesions in these areas (right vertical bar). Images are presented in neurological convention.

At baseline, the eighteen left frontoparietal stroke patients selected were submitted to clinical interview and neuropsychological assessment. The neuropsychological exams were administered by trained neuropsychologists and comprised the registration of sociodemographic information and the application of general cognitive abilities, episodic memory, and executive function neuropsychological tests (see details in Section 2.3). After the clinical and neuropsychological assessment, one patient was excluded due to the need of psychiatric medication for depression symptoms. On the day following these procedures, the 17 patients were examined with fMRI during the encoding of a face-name task (see details in Section 2.4.2) and off scan with a Face-Name Recognition Task (FNRT) (see details in Section 2.5) conducted by two different neuropsychologists and the biomedical staff from the Department of Radiology. After the first (baseline) fMRI exam, five patients were excluded from the study due to their incompatible work schedule. The 12 remaining left-sided stroke patients completed a baseline transfer and generalization ecological examination with 90 minutes of duration one week after the fMRI exam (see detailed description below in Section 2.6). Thereafter, during the following two weeks, they received three individual sessions of 90 minutes of duration with a two- or three-day interval between sessions of MST using face-name association strategy (see detailed description in Section 2.7). The posttraining transfer and generalization ecological examination were conducted two days after the third session. After one week of postintervention generalization session, they carried out the second posttraining fMRI acquisition and neuropsychological assessment of memory and executive functions on a separate day. One of the patients was excluded from the study due to pregnancy and did not perform the postintervention fMRI and neuropsychological exam. On the present study, we only used the behavior and image data of the remained 11 patients that completed all assessment and training stages. The details of all study phases are illustrated in Figure 1. Patients' clinical and sociodemographic information is shown in Table 1 and lesion maps are shown in Figure 2.

### 2.3. Neuropsychological Assessment of Episodic Memory and Executive Functions

The baseline neuropsychological assessment included measures of general cognitive abilities by IQ estimation (WAIS-III) calculated from the Vocabulary and Matrix Reasoning subtests [16, 17]. Episodic memory and executive function tasks were assessed at baseline and posttraining period and included the Revised Hopkins Verbal Learning Test (HVLT-R) [18], Revised Brief Visuospatial Learning Test (BVMT-R) [19], and Digit Span (WAIS-III) [16]. Parallel versions of the HVLT-R and BVMT-R were used to access episodic memory at baseline and posttraining phase. The executive function and attention domains were evaluated by the Victoria Stroop Test (VST) [20], Trail Making Test (TMT) [21], Modified Card Sorting Test (MCST) [22], and Phonemic (letters F, A, and S) and Semantic Verbal Fluency (animals) Tests [23].

### 2.4. fMRI Procedures

#### 2.4.1. fMRI Data Acquisition

Structural and echoplanar functional images for the whole brain were obtained on a Philips Achieva system 3T MRI scanner, using a 32-channel head coil. For blood oxygen level-dependent (BOLD) paradigm, T2∗-weighted echo planar (EPI GRE) images were acquired for each task run (TR = 3000 ms, TE = 30 ms, 40 slices, 3 mm slice thickness, FOV 240 mm, and matrix 80 × 78, with 135 volumes per run) and for the resting-state condition (*R* = 2000 ms, TE = 30 ms, 41 slices, 3 mm slice thickness, 0.3 mm of gap between slices, FOV 240 mm, and matrix 80 × 80, 200 volumes). Also, T2-weighted FLAIR images and T1-weighted three-dimensional images (voxel size: 1 mm^3^) were obtained for lesion evaluation and registration with the functional data.

#### 2.4.2. Experimental Design and Stimuli

**Figure 3:**
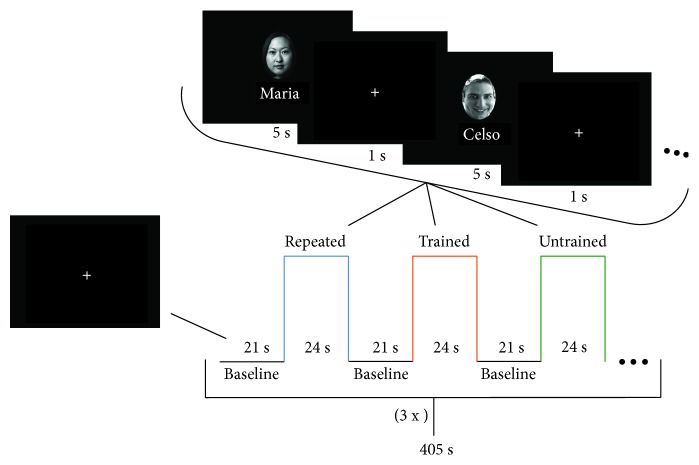
fMRI task experimental paradigm design of each run.

The same face-name associative encoding paradigm was administered at the baseline and posttraining fMRI exams based on a previous paradigm adapted from Hampstead and colleagues [11] and translated to Portuguese by Simon et al. [12]. The block design paradigm was composed by a baseline and three task conditions: repeated stimuli, trained stimuli, and untrained stimuli. The task was performed in 2 runs with 6 minutes and 45 seconds each, with 3 repeated condition blocks, 3 trained face-name pairs condition blocks, 3 untrained face-name condition blocks, and 9 baseline blocks per run, as shown in Figure 3. The baseline blocks lasted 21 s, where subjects saw a fixation cross, whereas the active blocks were presented during 24 s, where the subjects memorized 4 face-name pairs. On the active blocks, each face-name pair was presented for 5 s and followed by a 1 s interval (fixation cross). In the repeated condition, 2 face-name pairs were presented, while on the trained and untrained conditions, a total of 48 new face-name pairs were presented only one time during the scanning session. Although the order of active blocks was fixed, their stimuli were presented once in random sequence into each block to avoid the order bias. The paradigm was presented with E-Prime 2.0 software (Psychology Software Tools Inc., USA). Before the scanning session, each subject performed a brief face-name training with the repeated face-name pairs to ensure the comprehension of the task and the familiarization with the repeated stimuli. Also, they received explicit instructions to try to memorize each name associated with its respective face in silence during the scanning session. No other response was required since this could distract the subject from the implementation of the trained memory strategies.

### 2.5. Face-Name Recognition Task and Spontaneous Strategy Implementation Inquiry

All patients completed the Face-Name Recognition Task (FNRT) involving all 48 face pairs 20 minutes after the fMRI acquisition at the baseline and after MST. The patient had to identify which of the four-name choice displayed was the true pair of the face previously seen during the paradigm. The four names displayed included the target name, 2 familiar foils (i.e., names of other face pairs presented during the exam), and 1 novel name. This design was chosen to reduce the chance level performance to 25% and to enhance the need for recollection rather than mere familiarity. After performing the FNRT, patients answered a Spontaneous Strategy Implementation Inquiry (SSII) on which they rated how often they had used four of the possible strategies to study the face-name pairs. The SSII comprised three questions of memory encoding strategies cited in an earlier study [24]: (1) a verbal repetition strategy question (How often did you repeat the names of each face to yourself while you were seeing it on the scan?), (2) a visual inspection question (How often did you study the physical features of each face while you were seeing it on the scan?), and (3) an autobiographical association question (How often did you associate the face or the name with persons you already know?). The last question included the (4) MST implementation (How often did you associate a salient physical feature of the faces with their respective names and created a nickname to better remember them?). The participants' responses were transformed into numerical values using a five-point scale: Never (1), Rarely (2), Sometimes (3), Usually (4), and Always (5).

### 2.6. Transfer and Generalization Ecological Examination

Before and after MST, all patients completed the Multifactorial Memory Questionnaire (MMQ) [25, 26], the Brief Face-Name Questionnaire (BFNQ), and parallel versions of the Strategy Use Task (SUT) [12]. The BFNQ has four questions in which the patients must estimate the frequency of their memory difficulties and how often they used memory strategies to (1) remember names of persons they met and (2) remember faces of new ones they recently met on the last two weeks. The ratings were converted into numerical values using a five-point scale: Never (1), Rarely (2), Sometimes (3), Usually (4), and Always (5).

The SUT was developed by Simon et al. [12] in order to access the frequency and successful memory encoding while participants implement the MST. In the current experimental task, participants were asked to memorize the names of 12 new face-name pairs presented for 15 s each. After a 20 min interval, the faces were presented again one by one and the participants had to recall their appropriate name (cued recall). Subsequently, a three-choice recognition task including the target name, a name from a different pair within this same task, and a novel name was applied. After each response, participants were asked if they used any strategy to remember the name. If their responses included any associative link between face and name, one point was provided.

### 2.7. Face-Name Memory Strategy Training

The MST was based on the modified version of Biographical Information Module from the Ecologically Oriented Neurorehabilitation of Memory (EON-MEM) program [27] adapted by Hampstead et al. [13] and translated and validated to our language and culture by Simon et al. [12]. Participants were trained with 24 face-name pairs, divided across three training sessions (12 pairs in each of the first two sessions followed by a revision of all the 24 pairs on the third session). During the MST, participants were instructed to perceive a salient facial feature (visual cue) for each face-name pair. Then this physical feature was associated with a nickname that often rhymed with the actual name (verbal cue), while they created mental images that exaggerated and emphasized the facial feature. Thereafter, they were required to first recall the facial feature, then the nickname, and finally the corresponding name. These three strategy steps were recalled for each face-name pair until the participant accurately remembered them on three consecutive trials or on up to ten training trials per stimulus. Once all training trials had been completed for each 12 face-name pairs, the trained face-name pairs were reviewed using the same three strategy steps (same day review). On the next training session, participants first reviewed the 12 face-name pairs trained on the previous session (delayed review), and then they were trained on the 12 novel face-name pairs followed by the same day revision. The final training session started with a delayed review of 12 face-name pairs trained on previous session followed by a final revision of all 24 face-name pairs trained. Also, participants completed an ecological “generalization step” at the end of each session in order to apply the strategy learned with real people. Participants were asked to choose somebody whose name they had trouble recalling or had forgotten at least once. Participants were instructed to imagine the face of the person in detail, to describe it out, and then they tried to create their own associations with the help of the therapist.

### 2.8. Data Analysis

#### 2.8.1. Behavioral Analysis

We used JASP statistical package [28] to analyze all behavioral and cognitive measures including neuropsychological tests, the FNRT, the FNMGT, and perceived strategy questionnaires. The raw scores of these measures were analyzed using the one-tailed paired Student *t*-test or Wilcoxon signed-rank test to compare the pre- and postintervention outcomes. The nonparametric tests were adopted to ordinal variables or when the variable failed on the Shapiro-Wilk test. Cohen's *d* were used to estimate effect size of *t*-tests; for Wilcoxon tests, effect sizes were estimated by matched rank biserial correlation (*r*_rb_). Differences between conditions were considered significant if *p* < 0.05 after FDR-adjusted *p* value correction.

#### 2.8.2. fMRI Analysis

FMRI data analysis was performed with FSL 5.06 [29, 30]. FMRI data were preprocessed with the following steps: image realignment, slice timing correction, spatial smoothing with a 5 mm full width at half maximum Gaussian kernel, and a high-pass temporal filtering, to filter oscillation periods longer than 150 s. Images were registered into MNI152 6th generation template using a lesion mask created by a neuroscientist and revised by a radiologist with more than 10 years of experience. In this process, the lesion mask from each subject was used to remove the weight of the injured region in the T1 structural image linear registration onto MNI152 template. Each functional image was first registered to the patients' T1 image and then transformed into MNI space.

Then, for the first level analysis, a GLM model was used to estimative the BOLD response associated with each type of task: repeated faces, trained faces, and untrained faces. In this model, 6 variables of movement were used as covariate to reduce motion artifacts. At a higher level, there is a pretraining difference between novels (combination of trained and untrained blocks, given that prior to the training they should be equivalent) and repeated images. Also, the training effects (post- versus pretraining) were evaluated with paired tests comparing each pair of tasks. The trained > repeated along with trained > untrained analyses highlight the training-specific-induced changes, while the untrained > repeated comparison explores possible generalization (nonspecific) effects of the training.

To explore the relationship between training effects on brain activity and the improvement in memory performance, we calculated a percentage of performance using the pretraining score as baseline, by dividing the post- by pretraining score. This was calculated separately for trained and untrained images, and then the difference between these tasks was used as variable added to the trained > untrained higher level analysis, to provide the areas with BOLD signal task-induced changes correlated with the evolution of performance caused by the training. All the statistical images were thresholded using Gaussian random field-based cluster inference (a familywise error rate control method) with a threshold of *Z* > 2.3 at the voxel level and a corrected cluster significance threshold of *p* < 0.05.

## 3. Results

### 3.1. Cognitive and Behavioral Changes after MST

Cognitive tasks and self-perception questionnaire results on MST pre- and posttraining are shown in Table 2. In the neuropsychological tests, there were significant effects only on delayed memory scores of the HVLT-R (*t* = −10.035, FDR-corrected *p* = 0.0032, and *d* = −1.509). Regarding the off-scan recognition task, there were significant performance improvements on the total of face-name pairs accurately recognized (FNRT total; *t* = −10.035 , FDR-corrected *p* = 0.000016, and *d* = −3.026) and on the correct recognition of trained face-names (*t* = −9.341, FDR-corrected *p* = 0.000016, and *d* = −2.816). Moreover, significant improvements were observed on SUT recognition (*w* = 0.000, FDR-corrected *p* = 0.032, and *r*_rb_ = −1.000), but not on other SUT scores, which did not survive the FDR correction. Also, there were no significant differences on self-reports of memory functioning (MMQ, SSII) except for BFNQ strategy for faces (*w* = 1.500, FDR-corrected *p* = 0.007, and *r*_rb_ = −0.955), although BFNQ improvement and strategy use for name were borderline significant after FDR correction, and strategy use had the same effect size, as the BFNQ strategy for faces.

### 3.2. Pretraining Brain Activations

**Figure 4:**
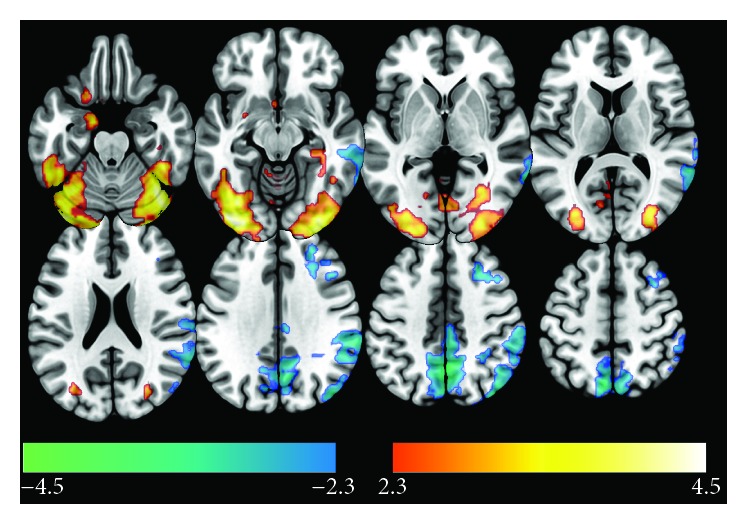
Pretraining activation. BOLD signal differences between novel versus repeated face-name associations. Voxel threshold *Z* > 2.3 and cluster-corrected *p* value < 0.05. Images are presented in neurological convention.

The pretraining fMRI results are presented in Table 3 and in Figure 4. The pretraining contrast between novel and repeated face-name pairs showed that novel pairs produced greater BOLD activations on the fusiform gyri, occipital cortex, inferior temporal gyrus, and cerebellum. However, these regions seemed to be active in both novel and repeated pairs, but with greater activation during novel face-name associations. Also, there were differences in the right amygdala, right hippocampus, and parahippocampal gyrus, due to their engagement for encoding of novel face-name associations.

On the other hand, the repeated versus novel contrast yielded significant differences in BOLD responses bilaterally in default-mode network (DMN) regions, such as the precuneus and inferior parietal areas (angular gyrus and supramarginal gyrus), and in superior division of the lateral occipital cortex, posterior areas of the right middle temporal gyrus, parietal operculum, planum temporale, and superior frontal and middle frontal gyrus. Most of these areas showed deactivations during novel condition, except for the frontal regions.

### 3.3. Posttraining Brain Activations

#### 3.3.1. Training-Specific Change Contrasts

**Figure 5:**
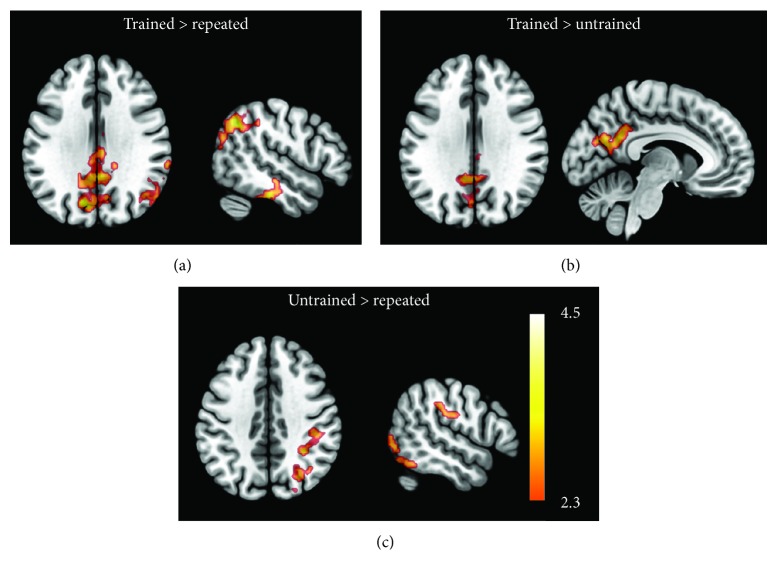
Training effects on brain signal. Comparison of post- versus pretraining BOLD responses to each type of face-name association: trained against repeated (a), trained against untrained (b), and untrained against repeated (c). Voxel threshold *Z* > 2.3 and cluster-corrected *p* value < 0.05. Images are presented in neurological convention.

**Figure 6:**
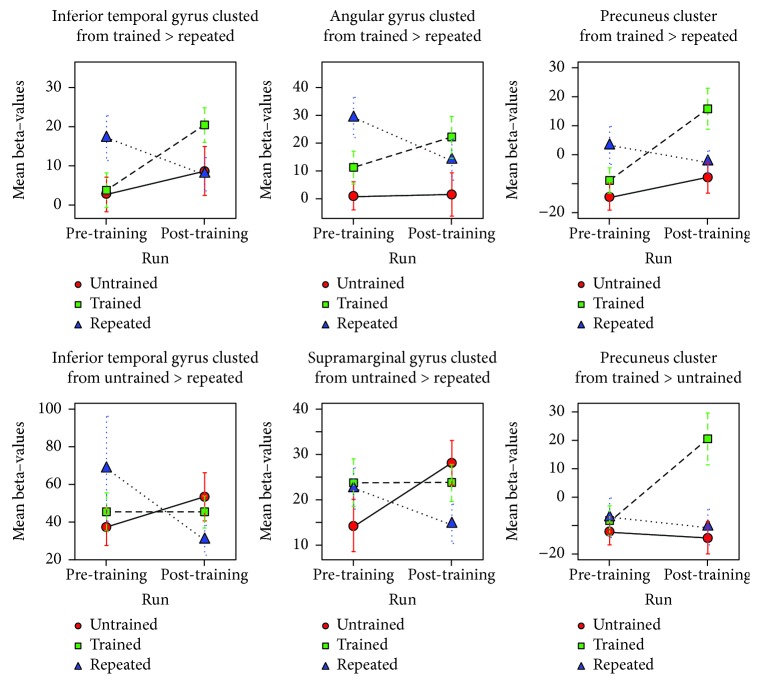
Mean beta value plots. Mean beta values from each condition (trained, untrained, and repeated) in each cluster with significant training effect (specific and nonspecific).

Trained (post > pre) > untrained (post > pre) face-name contrast revealed differences on the medial parietal areas (posterior cingulate cortex and precuneus cortex) and on the occipital cortices (left cuneus, left intra- and supracalcarine cortices), as described in Table 4 and shown in Figure 5(b). A detailed look at the beta values from the first level indicates that these differences are associated with an increased posttraining BOLD response in these regions only during the encoding of trained faces (Figure 6) and this pattern of enhancement of BOLD signal was observed in 10 of 11 patients.

Moreover, similar results were found in trained (post > pre) > repeated (post > pre) face-name contrast (Table 4; Figure 5(a)). Aside from the medial parietal areas (right posterior cingulate cortex and bilateral precuneus cortex) found on the previous contrast, there were significant training effects on the occipital cortices (left cuneus, lateral occipital areas, and intra and supracalcarine cortices), on the right inferior parietal cortex, and on the right temporal cortex (inferior and middle temporal gyrus, temporal fusiform cortex, and posterior division of superior temporal gyrus). The examination of the outcomes of the first-level analyses revealed that this result was also related to consistent higher increases in activation after MST for trained stimuli (Figure 6), as this was observed in all these areas for at least on 8 of the 11 patients. Additionally, the BOLD signal decreased on the right superior division of the lateral occipital cortex, angular gyrus, supramarginal gyrus, and on the posterior division of superior temporal gyrus for repeated stimuli in 8 of the 11 patients.

#### 3.3.2. Nonspecific Changes

The untrained versus repeated face-name contrast yielded positive BOLD responses on the right lateral areas of the parietal (superior parietal lobule, supramarginal gyrus, anterior intraparietal sulcus, and postcentral gyrus), temporal (inferior temporal gyrus, occipital fusiform gyrus), and occipital cortices (lateral occipital cortex), as described in Table 4 and illustrated in Figure 5(c). These regions seem to be engaged in all conditions both before and after the training. However, the untrained face-name pairs induced a higher activation in the posttraining run, while the repeated ones had smaller posttraining activation (Figure 6). Further examination of the values extracted from the first-level analyses confirmed that these effects were consistent, especially in the supramarginal gyrus, anterior intraparietal sulcus, superior parietal cortex, and lateral occipital cortex, where this pattern of change in BOLD response was consistent across 9 of 11 patients.

### 3.4. Brain Activations and Performance Change Correlations after Memory Strategy Training

**Figure 7:**
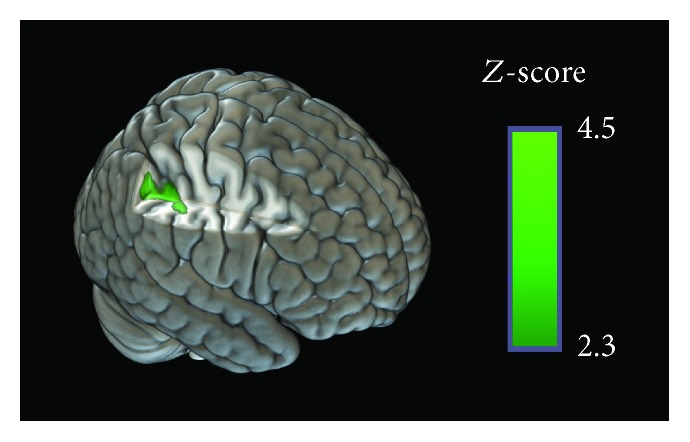
Correlation between brain activity and FNRT proportional posttraining performance. Region of brain activity significantly correlated to improvement of performance in Face-Name Recognition Task, given by the posttraining score divided by pretraining score. Voxel threshold *Z* > 2.3 and cluster-corrected *p* value < 0.05.

There is a positive correlation between better trained versus untrained proportional posttraining performance on FNRT (posttraining scores divided by the pretraining scores) and higher differences in BOLD responses in trained (post > pre) versus untrained (post > pre) contrast on the right supramarginal gyrus, angular gyrus, superior parietal lobule, anterior intraparietal sulcus, and lateral occipital cortex (superior division). This cluster seems to be engaged in all conditions (even repeated faces), before and after MST. Brain activation map correlated with gains in performance in FNRT after MST is shown in Figure 7.

## 4. Discussion

In the present study, we investigated the behavior effects and the neural correlates of MST using face-name strategy and fMRI in patients with left stroke lesions. Previous studies have indicated that CT can produce a restorative mechanism by facilitating the residual functioning of a certain brain regions or the recruitment of other related brain areas leading to compensation mechanism [12, 31–34]. In stroke, computerized cognitive training has been associated with increases in functional connectivity of the hippocampus with prefrontal and parietal areas and enhancement in memory and attention performance [35]. However, the previous evidence available [35, 36] of effect of CT on the neural substrates of stroke patients was not focused on the effect of MST during an active memory encoding task on fMRI. Evidence of efficacy of memory rehabilitation on stroke patients is still under debate due to its heterogeneous nature and spontaneous memory recover over time [37]. Our study used individual sessions to teach stroke patients a single specific intervention to reduce the confusion caused by the implementation of multiple mnemonic strategies [11, 13, 14] and encourage them to apply the trained strategies in daily life situations. Our case series study demonstrated brain-behavior improvements associated with MST which we will discuss in detail on the next sessions.

### 4.1. Behavior and Cognitive Changes

The MST for face-name associative encoding applied during 3 individual sessions of intense specific training seemed to beneficiate left-sided stroke patients since they demonstrated significant enhancement on recognition performance of trained stimuli and on total of correct FNRT. Also, stroke patients demonstrated increased recognition on the SUT tasks, a set of other untrained face-name pairs not related to the fMRI task which could imply the effective learning and application of the MST. So, despite the reduced number of sessions and different clinical population, our results are in line with the two previous studies that applied the same MST in MCI population [11–13].

Another interesting finding was related to far-transfer effects measured by the episodic memory tests using parallel versions of HVLT-R and BVMT-R and metamemory questionnaires. We found significant improvements on a delayed verbal memory recall measure (HVLT-R) after training. The improvement on these measures could be related to the semantic cue created during the MST sessions where the salient face feature was classified with a verbal cue that rhymed with the proper name of the face presented. Since semantic clustering strategies can be associated with the improvement of performance on this test [32, 33, 38], we hypothesize that their performance was beneficiated partially due to the strategic training.

Regarding the self-report questionnaires, stroke patients reported significant improvements on strategy for learning new faces. They did not report significant improvements in strategy use during the fMRI task (SSI) or global metamemory function measured by MMQ. Although the enhancement on MMQ scores was previously reported by Simon et al. [12] using the same MST for face-name associative encoding on MCI patients, the lack of significant improvement on MMQ scores and other self-report measures in our study may be due to the poor self-awareness of the stroke patients regarding their memory function [39]. Aben et al. [40] found that stroke patients' perception of their memory function is predicted by their memory self-efficacy rather than their true memory performance on neuropsychological tests. Moreover, they found that left-sided stroke patients had worse perception of their memory than other stroke patients. In addition, other memory rehabilitation studies with acquired brain-injured patients demonstrated that memory function improvements are perceived more by other informants, like relatives, than the patients themselves [41, 42]. We did not assess other informants' perception of stroke patients' memory function. The lack of self-awareness and reduced number of participants may have contributed to the underestimation of the self-report questionnaires of memory function and strategy memory use. Nevertheless, despite the underestimation of memory performance by the stroke patients, their report of a better memory strategy to remember new faces along with memory improvement on SUT recognition task supports the beneficial effect of MST for face-name associative encoding in this population.

### 4.2. Specific Brain Change Results Related to MST

Recent evidence showed that MST leads to distributed changes on a brain function network organization on visual, medial, temporal, and default-mode networks (DMN) related to a better memory performance in normal subjects [43]. On MCIs, MST for face-name association leads to enhanced brain activations on DMN regions including the medial, frontal, and parietal cortices, lateral temporal cortex, and angular gyrus [11]. We found quite similar patterns of BOLD response with increasing activations on most of these areas except for more anterior brain areas. We also found increased activation on the left cuneus, bilateral lateral occipital cortex, right superior temporal gyrus, and on right temporal fusiform cortex. Similar to Hampstead et al. [11], we found enhanced activations on the posterior cingulate cortex (PCC) which establishes rich reciprocal connections with DMN regions and interacts with the medial temporal lobe memory system [44–46]. The PCC seems to be responsible for the integration of self-referential processing with other cognitive processes such as episodic retrieval, emotional processing, visual imagery construction, and awareness [47, 48]. Moreover, the PCC is recruited during the recollection processing on recognition tasks [49] and on retrieval of real episodic memories rather than constructed ones [50]. This implies that the involvement of this area after MST could be related to the recognition based on vividly remembering specific contextual details of trained stimuli and the retrieval of personal experiences during the training sessions, since this area was activated only for face-name pairs related to the MST sessions. Also, the enhanced activation of PCC in conjunction with the precuneus cortex could reflect the retrieval and implementation of mental imagery mnemonic strategy taught on the MST sessions since these areas have extensive reciprocal connections [51], are relevant to better memory performance [51, 52], and are highly activated after MST on normal subjects [33, 53, 54] and MCIs [11]. Finally, the enhanced activation on the precuneus and cuneus cortex could reflect the attentional demands and specific cue tracking during the recollection of the previously learned strategies, given that these areas are involved on controlled aspects of attention on a long-term memory search [55].

We also found an enhanced activation on the lateral temporal cortex which is involved on semantic memory retrieval [56]. This memory process has been engaged in episodic memory recall [57] possibly due to the constant use of conceptual knowledge in our daily experiences, which represents an “integration of episodic and semantic contents” [58, 59].

Contrasting with the previous reports of Hampstead et al. [11], we found a contralateral-enhanced activation on areas around temporoparietal junction on the right hemisphere after MST. This contralateral pattern of activation could represent a hemispheric compensation mechanism or could be a result of general reorganization of brain function due to the left ischemic lesion and that was already seen in acquired brain injury sample studies [34, 60]. However, it was possible to observe an enhancement on BOLD signal on these areas after MST that were not activated at the baseline period. There is some evidence that these regions, particularly the inferior parietal lobe area, mediate bottom-up attentional processes that are captured by a salient memory-relevant output [61, 62]. The inferior parietal lobe is thought to be responsible for the maintenance or representation of retrieved information, acting as an episodic buffer of a long-term memory [63]. Also, this region is suggested to be part of a hippocampal-parietal memory network due to its strongly correlated activity with the hippocampal formation and lateral temporal cortex regions [57, 64].

As we stated earlier, during the MST, patients learned a mnemonic strategy by selecting a salient feature of the face that could be semantically associated with its name integrating semantic contents to episodic memory and also to a memory schema. Schemas are frameworks of acquired knowledge that facilitates the assimilation of new related information, leading to a better retention of this information [65–67]. Application of schemas during experimental task seems to be mediated by the parietal cortex regions, particularly by the angular gyrus [68–70]. The angular gyrus (AG) seems to be responsible for binding the different contents into a schema within the parietal cortex [71]. Due to its location at the junction of visual, spatial, somatosensory, and auditory processing streams, the AG integrates all these sensory modality attributes in semantic and conceptual associations [57] facilitating memory encoding and retrieval [71]. The enhancement of BOLD signal on this area after MST may be due to the retrieval of the previously learned strategy.

### 4.3. Nonspecific Training Changes Related to MST

The enhancement of BOLD signal after training on the right lateral areas of the parietal and occipital cortex during the associative encoding of untrained face-name pairs, added to the BOLD signal decline for repeated face-name pairs, may reflect patients' attempts to generalize training strategies to untrained stimuli. Hence, practice effects on repeated images would result in decreases in BOLD signal, due to reexposure to the same stimuli seen in pretraining [11]. Also, we found a tendency of reduction in the BOLD response for trained faces that reinforces this hypothesis, suggesting distinct responses of BOLD signal for trained and untrained stimuli, a result that was not reported by the previous study using this paradigm [11]. MST yields increased BOLD signal response on the superior parietal cortex and on intraparietal sulcus. These regions were previously reported to be engaged in task involving the ordination, updating, and manipulation of items in working memory [72, 73]. Additionally, the intraparietal sulcus seems to be critical to spatial attention processing [74, 75]. Moreover, increased activations were found on supramarginal gyrus, which is recruited in phonological processing [73, 76, 77]. Similar effect was also found on superior division of the lateral occipital cortex, related to the processing of specific facial features after an MST [78, 79]. The enhanced activation on these areas may be the reflection of stroke patients' attempt to generalize the strategy previously learned to the untrained stimuli. The MST involves the selection and recombination of visual and verbal features engaging working memory processing.

### 4.4. Brain Changes Correlated with Increased Performance on FNRT

It is interesting to note that the improvement of performance on recognition task comparing trained and untrained stimuli was correlated with the activation of BOLD signal in trained (post vs. pre) > untrained (post vs. pre) contrast. These results were found in brain areas that presented higher posttraining activity, either for trained stimuli (as the AG) or for untrained stimuli (as the supramarginal gyrus, superior parietal lobule, and anterior parietal sulcus), which were previously described as multidomain parietal areas identified as “hubs” by a large-scale meta-analysis of fMRI studies [73]. Also, these regions seemed to have some level of activation in all conditions (pre- and posttraining, and even in repeated faces) corroborating with the idea of hubs of attentional processing and attempt of information integration that were present in all conditions. More specifically, the supramarginal gyrus was recruited by both bottom-up attention and phonological processing, while the AG was involved in automatic semantic retrieval and high confidence episodic retrieval. Finally, the anterior IPS was engaged on high executive demanding tasks, such as executive semantic processing, top-down attention, and working memory. Additionally, Humphreys and Lambon [73] demonstrated that the anterior IPS and the AG could both be positively engaged in a specific task, but the anterior IPS exhibited greater activation related to difficulty on semantic and visuospatial tasks. This may be the reason for the increased activation on anterior IPS, as previously seen on the encoding of untrained stimuli. It is possible that the creation and implementation of an MST in a brief interval of exposition of a face-name pair may be more demanding than remembering a previously learned schema. Moreover, these cognitive operations, schema identification, attentional control, and working memory processing, seem to be relevant to successful performance on the FNRT, given that increased activations were found in both areas (AG and anterior SPG) in conjunction with the superior parietal cortex [70, 72, 73] and lateral occipital cortex, including ventral visual areas which are important for facial feature processing [78, 79]. There is some evidence that activations on brain areas that are involved in the original processing of the stimuli during the encoding, like the lateral occipital cortex, support lasting memory representations [80–82].

It is important to notice that the performance index used in this analysis provides a measure of proportional improvement difference, in a way that subjects that had a poor performance in the pretraining run could show a significant improvement, even if their final posttraining FNRT score was not so high. This might be a more sensitive index for performance improvement due to the proper use of the strategy, possibly explaining the fact that we found significant correlation, even though there were no differences of training effects in brain activation between trained and untrained conditions in these parietal areas. Therefore, considering these correlation results together with the training effects observed in the brain activation differences, these areas seem to be relevant not only for the attempt of using the strategy but also for the memorization *per se* with the strategy (especially in the AG).

### 4.5. Study Limitations

Memory dysfunction after ischemic stroke could be influenced by several variables and associated with the disruption of other main cognitive processes, such as language or visuospatial processing, and could also be influenced by spontaneous recovery mechanisms. We tried to limit these variables by including nonaphasic chronic stroke patients with the left-hemisphere ischemic lesions and preserved hippocampus areas. This specific focus limited our patient selection due to multiple exclusionary factors; however, these criteria were important to focus on our research question and provided a more consistent sample, hence, the small size. Also, given this small sample size, a more permissive voxel level threshold of *Z* > 2.3 was adopted, while the current recommended value would be *Z* > 3.1 [83]. However, a more permissive approach allows a lower false-negative rate that should be also considered [84], especially for use in future meta-analyses. Also, the similarity of our results with a previous study [11] might indicate that these findings are less likely due to false-positive effects. Therefore, these results are preliminary and could contribute to further investigations with large samples and to future meta-analyses in order to further explore MST in patients with ischemic lesions. It is important to point out that we used a recollection performance measure to correlate with brain activation during the associative encoding of face-name paradigm, and our results suggest a correlation with effective encoding indirectly measured by the recognition task like other studies published with a face-name paradigm [11, 12]. In addition, we found robust behavioral results that may suggest far and near-transfer effects related to MST, and the neural changes were consistent in most of the subjects that participated in the study. These findings could be used as a basis for future studies with specific memory training.

## 5. Conclusions

Evidence of the efficiency of memory training on brain functioning of stroke patients is sparse [35, 36] and questioned by its heterogeneous nature and the mechanisms of spontaneous recovery over time [37]. Our study provided evidence of potential benefits of a specific mnemonic training on behavioral and brain functioning in the left-hemisphere chronic ischemic stroke patients. After the mnemonic training, patients demonstrated engagement of distributed neural networks that mediated memory functions like visual, temporal, parietal, and DMN areas in line with the previous studies [11, 12, 43]. Also, the performance improvement was associated with increases on the right contralesional areas including the superior parietal cortex, the supramarginal gyrus, the intraparietal sulcus, the angular gyrus, and the lateral occipital cortex. These brain regions are involved in the processing of cognitive operations, including attentional control, working memory, schema, and facial feature identification [70, 72, 73, 78, 79], and could be related to an efficient compensation mechanism. In addition, stroke patients showed possible near-transfer effects and evidence of immediate far-transfer effects related to the specific mnemonic training. They demonstrated an efficient learning and transfer of the face-name strategy encoding and showed improved memory performance, with better self-report in their capacity to learn novel faces. Together, these findings suggest that MST can promote positive effects on cognitive and brain functioning in the left-hemisphere stroke patients associated with recruitment of DMN, frontoparietal control network, and dorsal attention network areas as a possible compensation mechanism.

## Figures and Tables

**Table 1:** Sociodemographic and clinical characteristics of each of the left-sided stroke patients previous to MST.

Patient	Sex	Age	School years	Years since lesion	Etiology	Lesion size (cm^3^)	Lesion location on the left hemisphere
P1	M	20	12	2	MCA ischemia	10.63	FP, PreCG, PostCG, INS, & Pu
P2	M	29	13	4	MCA ischemia	23.99	IFG, PreCG, & INS
P3	F	30	11	1	MCA ischemia	20.48	FP & IFG
P4	F	36	15	5	MCA ischemia	19.76	IFG, MFG, PreCG, & INS
P5	F	43	11	3	MCA ischemia	67.09	IFG, PreCG, PostCG, NCR, & Pu
P6	F	44	11	12	MCA ischemia	89.61	IFG, MFG, SFG, PreCG, PostCG, INS, & SPL
P7	M	51	11	2	MCA ischemia	61.18	IFG, MFG, PreCG, PostCG, STG, & IPL
P8	F	45	11	3	MCA ischemia	17.28	PreCG, PostCG, & INS
P9	F	48	15	4	ACA ischemia	8.75	SFG, PreCG, & Cg
P10	F	55	11	10	MCA ischemia	12.73	PreCG & PostCG
P11	M	57	11	3	MCA ischemia	13.20	Superior part of PreCG & PostCG

M: male; F: female; MCA: middle cerebral artery; FP: frontal pole; IFG: inferior frontal gyrus; MFG: middle frontal gyrus; SFG: superior frontal gyrus; PreCG: precentral gyrus; PostCG: postcentral gyrus; STG: superior temporal gyrus; SPL: superior parietal lobule; INS: insula; Cg: cingulate cortex; NCR: nucleocapsular region; Pu: putamen.

**Table 2:** MST effects on cognitive and behavioral performance of left-sided stroke patients.

	Pretraining	Posttraining	FDR-corrected *p* value	Effect size
	Mean ± SD	Mean ± SD		
*Neuropsychological measures*				
HVLT-R immediate learning	22.273 ± 6.182	23.455 ± 7.568	0.296	*d* = −0.300
HVLT-R delayed recall	6.455 ± 3.236	8.818 ± 2.714	0.003∗	*d* = −1.509
BVMT-R immediate learning	22.182 ± 7.427	22.636 ± 9.532	0.534	*d* = −0.063
BVMT-R delayed recall	8.182 ± 3.737	8.636 ± 2.803	0.382	*d* = −0.210
Digit span forward	6.182 ± 1.537	6.545 ± 1.695	0.368	*d* = −0.232
Digit span backward	3.727 ± 1.489	4.000 ± 1.000	0.447	*r*_rb_ = −0.576
TMTA	60.818 ± 3.9656	53.818 ± 34.251	0.228	*d* = 0.386
TMTB	144.182 ± 123.723	133.455 ± 77.137	0.572	*r*_rb_ = 0.030
VST-I	30.727 ± 26.627	28.273 ± 12.681	0.759	*r*_rb_ = −0.152
VST-II	44.273 ± 42.441	34.455 ± 22.138	0.534	*r*_rb_ = 0.091
VST-III	61.909 ± 61.800	47.000 ± 31.458	0.176	*r*_rb_ = 0.273
SVF	12.636 ± 4.632	12.273 ± 3.690	0.759	*d* = 0.148
PVF	22.545 ± 12.226	25.091 ± 13.736	0.106	*d* = −0.639
MCST	4.273 ± 2.005	4.364 ± 1.629	0.534	*r*_rb_ = −0.052
*Off-scan measures*				
FNRT—total	20.727 ± 7.617	32.636 ± 7.075	0.00002∗	*d* = −3.026
FNRT—trained stimuli	9.818 ± 4.215	19.182 ± 4.191	0.00002∗	*d* = −2.816
FNRT—untrained stimuli	9.091 ± 3.986	11.364 ± 4.478	0.108	*d* = −0.603
SSI verbal repetition	3.273 ± 1.348	3.818 ± 1.250	0.296	*r*_rb_ = −0.455
SSI visual inspection	3.636 ± 1.120	4.273 ± 0.786	0.226	*r*_rb_ = −0.636
SSI autobiographical association	3.818 ± 0.982	3.455 ± 1.293	0.773	*r*_rb_ = −0.485
SSI face-name association	2.818 ± 1.401	4.000 ± 1.612	0.172	*r*_rb_ = −0.515
*Ecological and generalization measures*				
SUT delayed recall	1.682 ± 3.272	2.545 ± 3.082	0.131	*r*_rb_ = −0.889
SUT delayed recall strategy	1.182 ± 3.231	2.409 ± 3.018	0.106	*r*_rb_ = −0.929
SUT recognition	4.227 ± 5.182	4.587 ± 5.430	0.032∗	*r*_rb_ = −1.000
SUT recognition strategy	1.455 ± 3.334	2.955 ± 3.618	0.080	*r*_rb_ = −0.945
MMQ contentment	35.091 ± 16.604	41.545 ± 13.873	0.296	*r*_rb_ = −0.348
MMQ ability	43.909 ± 13.111	50.545 ± 13.133	0.932	*d* = −0.489
MMQ strategy	27.909 ± 10.728	25.091 ± 10.849	0.845	*d* = 0.288
BFNQ—name difficulties	3.182 ± 1.079	2.636 ± 1.120	0.053	*r*_rb_ = −0.364
BFNQ—face difficulties	2.091 ± 1.044	2.000 ± 0.894	0.534	*r*_rb_ = −0.879
BFNQ—name strategy	4.182 ± 3.093	7.182 ± 3.027	0.055	*r*_rb_ = −0.955
BFNQ—face strategy	4.455 ± 2.697	7.455 ± 1.440	0.045∗	*r*_rb_ = −0.955

^∗^Significant differences (*p* < 0.05) after FDR *p* value correction. *r*_rb_: matched rank biserial correlation; *d*: Cohen's *d*; HVLT-R: Revised Hopkins Verbal Learning Test; BVMT-R: Brief Visuospatial Learning Test; VST: Victoria Stroop Test; TMT: Trail Making Test; MCST: Modified Card Sorting Test; PVF: Phonemic Verbal Fluency Test; SVF: Semantic Verbal Fluency; FNRT: Face-Name Recognition Task; SSII: Spontaneous Strategy Implementation Inquiry; MMQ: Multifactorial Memory Questionnaire; BFNQ: Brief Face-Name Questionnaire; SUT: Strategy Use Task.

**Table 3:** Pretraining significant clusters and peak MNI coordinates.

Regions within cluster	*X*	*Y*	*Z*	Cluster *P*	Number of voxels
*Novel > repeated*					
L lateral occipital cortex; L fusiform gyrus; L occipital pole; lingual gyrus; L cerebellum	-40	-86	-12	2.67*E* − 18	5421
R lateral occipital cortex; R fusiform gyrus; R inferior temporal gyrus; R occipital pole; R cerebellum	32	-88	-6	4.81*E* − 17	4906
L amygdala; L hippocampus and L parahippocampal gyrus; L fronto-orbital cortex	-16	-6	-20	0.0083	507
*Repeated > novel*					
R lateral occipital cortex (SD); R middle temporal gyrus; R angular gyrus; R supramarginal gyrus; R parietal operculum; R planum temporale	42	-78	40	2.12*E* − 13	3513
L/R precuneus cortex; R cingulate gyrus; L/R lateral occipital cortex (SD)	4	-56	40	9.77*E* − 12	2931
R middle frontal gyrus; R superior frontal gyrus	36	14	50	0.00226	618

SD: superior division; L: left; R: right.

**Table 4:** Posttraining significant clusters and peak MNI coordinates.

Regions within cluster	*X*	*Y*	*Z*	Cluster *P*	Number of voxels
*Trained > untrained*					
R/L precuneus cortex; L cuneal cortex; R/L posterior cingulate gyrus; supracalcarine cortex; intracalcarine cortex	-4	-66	22	2.98*E* − 06	1080
*Trained > repeated*					
R/L posterior cingulate gyrus; R/L precuneus cortex; L cuneal cortex; L lateral occipital cortex (SD); L intracalcarine cortex; L supracalcarine cortex	2	-40	44	1.82*E* − 11	2780
R angular gyrus; R lateral occipital cortex (SD); R supramarginal gyrus; R superior temporal gyrus (PD)	52	-58	38	8.00*E* − 05	913
R inferior temporal gyrus (PD); R temporal fusiform cortex (PD); R middle temporal gyrus	56	-36	-30	0.00939	486
*Untrained > repeated*					
R lateral occipital cortex (SD); R superior parietal lobule; R supramarginal gyrus (ID); R anterior intraparietal sulcus; R postcentral gyrus	26	-82	34	1.43*E* − 06	1166
R lateral occipital cortex (ID); R inferior temporal gyrus; R occipital fusiform gyrus	62	-54	-20	0.00632	457

## Data Availability

The data used to support the finding of this study are included within the article.
